# Molecular characterization of *Borrelia *strains isolated from ticks in Vojvodina

**DOI:** 10.1186/1756-3305-7-S1-P18

**Published:** 2014-04-01

**Authors:** A Potkonjak, S Savić, E Ruzić-Sabljić, V Vračar, B Lako, A Jurišić, A Petrović, D Rajković

**Affiliations:** 1 Department of Veterinary Medicine, Faculty of Agriculture, University of Novi Sad, Novi Sad, Serbia; 2 Scientific Veterinary Institute "Novi Sad", Novi Sad, Serbia; 3 Institut of Microbiology and Immunology, Faculty of Medicine, University of Ljubljana, Ljubljana, Slovenia; 4 Department of Environmental and Plant Protection, Faculty of Agriculture, University of Novi Sad, Novi Sad, Serbia

## 

*Borrelia burgdorferi *sensu lato (s.l.) complex represents a group of different types of spirochete that are present globally, which causes Lyme borreliosis. The total number of types is still not final because new genetic and antigenic isolates are still being described. The clinical picture of Lyme borreliosis in people is polymorphic and is characterized with symptoms similar to flu syndromes together with erythema migrans, rheumatologic, cardiologic and neurological complications. Apart from people, dogs, horses, bovines and sheep can suffer from Lyme borreliosis. In Europe, the most important vector transmitting Lyme disease is the tick *Ixodes ricinus*. A description of different species of *Borrelia *in ticks has opened a completely new field of investigating the ecology of Lyme borreliosis. It is especially important to investigate a connection between different types of *Borrelia*, ticks as vectors and vertebrates as the reservoirs on various geographical localities.

The aim of the work is to isolate *Borrelia *species from the collected ticks *I. ricinus *from the geographical territory of Vojvodina, and to do a molecular characterization of the isolated strains of *Borrelia*.

A total of 12 tick pools of the type *I. ricinus *were cultivated in Barbour-Stoenner-Kelly-H medium with additional antibiotics and subcultivated into a modified Kelly-Pettenkofer/Preac-Mursic medium, until a clear culture is reached. For the molecular characterization of isolated strains of *Borrelia*, molecular methods *Mlu*I-LRFP and real-time PCR for *hbb *gene were used.

In this research, out of 12 pools of ticks species *I. ricinus *3 strains of *Borrelia *from the *B. burgdorferi *s.l. complex were isolated. All three isolates of *Borrelia *from ticks of the tick species *I. ricinus *from the territory of Vojvodina were identified as *Borrelia afzelii *by applying molecular methods (*Mlu*I-LRFP and real-time PCR for *hbb *gene). By applying *Mlu*I-LRFP all three isolated strains of *B. afzelii *were characterized as a subtype Mlal. In this research, like in the previous researches, we have not proved the presence of a pathogenic species *Borrelia spielmanii *in ticks of the species *I. ricinus*.

## Funding

This research was supported by Provincial Secretariat for Science and Technological Development, Autonomous Province of Vojvodina, Republic of Serbia (grant numbers: 114-451-3406/2013-04 and 114-451-3638/2012-01).

